# Anterior Sectional Twin Bracket Appliance – Innovative Use for Correction of Single Tooth Crossbite: A Case Report with Biomechanics

**DOI:** 10.5005/jp-journals-10005-1286

**Published:** 2015-04-28

**Authors:** Raj Kumar Verma, Pradeep Raghav, Munish C Reddy, Ritika Kanwal

**Affiliations:** Senior Lecturer, Department of Orthodontics, Subharti Dental College Meerut, Uttar Pradesh, India; Professor and Head, Department of Orthodontics, Subharti Dental College Meerut, Uttar Pradesh, India; Professor, Department of Orthodontics, Subharti Dental College Meerut, Uttar Pradesh, India; Postgraduate Student, Department of Orthodontics, Subharti Dental College Meerut, Uttar Pradesh, India

**Keywords:** Single tooth crossbite, Sectional appliance, Biomechanics.

## Abstract

Anterior sectional twin bracket appliance (ASTBA) is a sectional mechanism that involves two brackets on upper central incisors. This appliance is previously been used for correction of rotated incisors and midline spacing. But, detail biomechanics for single tooth crossbite correction is not previously explained. Here, in this article, we are presenting a detailed biomechanics of ASTBA for anterior single tooth crossbite correction along with case report.

**How to cite this article:** Verma RK, Raghav P, Reddy MC, Kanwal R. Anterior Sectional Twin Bracket Appliance– Innovative Use for Correction of Single Tooth Crossbite: A Case Report with Biomechanics. Int J Clin Pediatr Dent 2015;8(1): 66-69.

## INTRODUCTION

Anterior sectional twin bracket appliance (ASTBA)^1^ is a sectional mechanism that involves two brackets on upper central incisors. Maxillary central incisors typically are situated in their sockets in such a way that their occlusal contour follows the normal arc of the maxillary dentition.^2^ However, in some reported cases of American Indians, the distal margins of the incisors are rotated in a labial or lingual direction. Lingual rotation has been termed counter winging by Dahlberg,^3^ whereas labial rotation is simply winging. This unusual rotation may lead to edge to edge bite or anterior crossbite of one or both maxillary central incisors. Anterior crossbite of single tooth affects around 4% of schoolchildren. This rotated incisor could be a reason for occlusal trauma to mandibular incisors, which may get displaced labially out of the arch and show gingival recession and mobility of affected tooth. Early correction is needed in these cases for esthetics, integrity of dental arch, normal growth of both jaws and proper oral functions. Crossbite of local origin can be treated by both removable and semifixed appliances. Removable appliances include upper removable appliance with Z spring and posterior bite block, and semifixed appliance used is 2 × 4 appliance which is comprised of molar bands with tubes on maxillary first molars and brackets on four maxillary incisors.^3^ However, they have certain limitations and disadvantages. Removable appliance with Z spring requires a high level of patient cooperation and takes longer time than fixed appliances to correct incisor crossbite. Also, this is not very effective in complete correction of rotation of incisor.^4^ Injudicious use of 2 × 4 appliance in mixed dentition stage may damage the roots of developing lateral incisor that are in close proximity of developing crowns of permanent canine.

In this article, we present an innovative use of ASTBA for correction of anterior single tooth crossbite along with biomechanics and case report.

## BIOMECHANICS

Anterior sectional twin bracket appliance has been previously successfully used for correction of bilaterally winged incisors^5^ by reciprocal anchorage. Tanaka et al^6^ have used this concept for midline diestema closure. However, there are certain problems for consideration before use of anterior sectional wire appliance in single tooth crossbite correction. One is disocclusion of dentition and another is anchorage requirement. Disocclusion beyond freeway space is required for labial movement of upper central incisor. Without anchorage preparation, there are chances that healthy incisor can rotate as a result of anchorage loss. We can address these two issues with one solution that is modified posterior bite plane. The modification incorporated is that it should be extended in the palatal area, especially adjacent to normally placed incisor in whole lingual region and distolingual portion of rotated tooth.

**Fig. 1:**
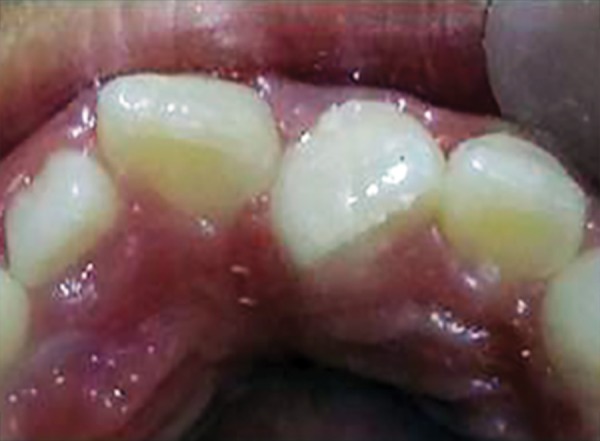
Mesiolingually rotated maxillary left central incisor (21)

**Fig. 2:**
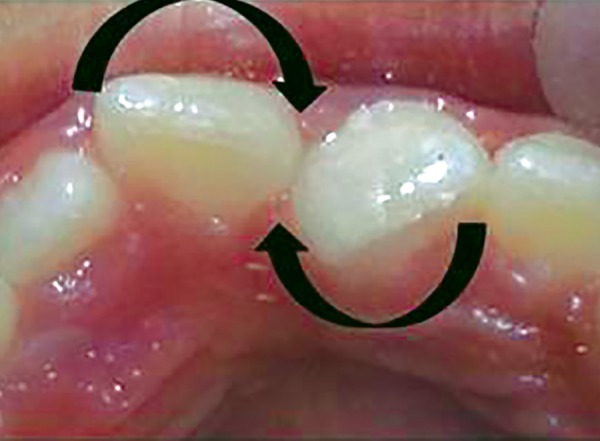
Diagrammatic representation of possible rotation of incisors

**Fig. 3:**
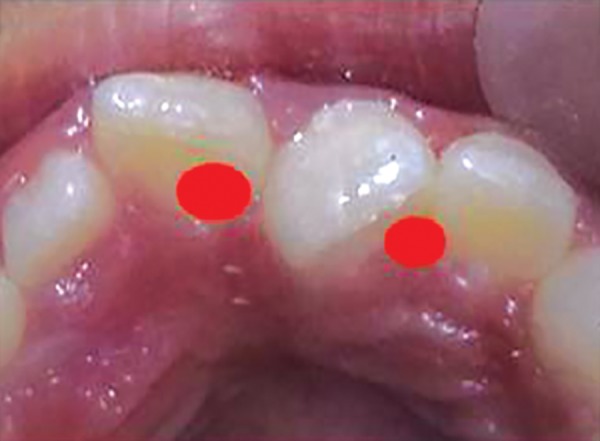
Critical areas to be covered with acrylic to prevent unfavorable rotation

**Fig. 4:**
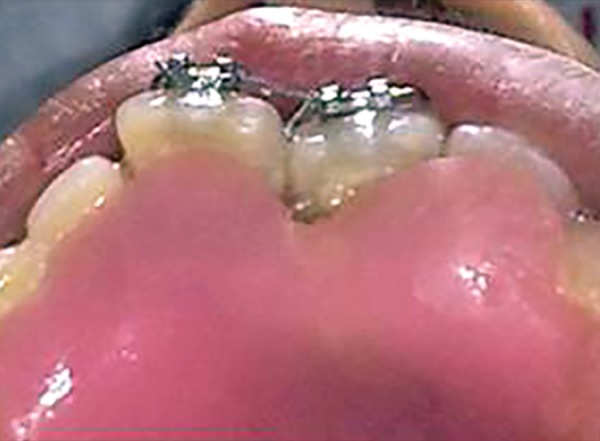
Properly extended bite plate in patient’s mouth along with partial correction of incisor

**Fig. 5:**
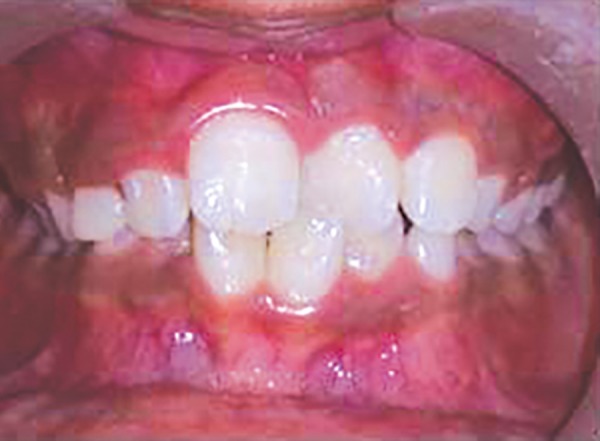
Intraoral frontal view

For example, in given case (Fig. 1), 21 was mesio-lingually rotated. When try to apply corrective forces on 11 and 21 by twin brackets and NiTi wire, 21 will come in alignment but it will also affect 11 and 11 will tend to rotate in mesiolingual direction (Fig. 2). Figure 3 shows critical areas to be covered with acrylic to prevent unfavorable rotation of 11. Figure 4 shows properly extended bite plate in patient’s mouth along with partial correction of incisor. In some cases where the rotated incisor is intruded, we may be required to extend the bite plate to cover the incisal edge to prevent its intrusion (Fig. 5). Recommended wear is removable use of bite plane along with twin brackets; however, we may need to cement the posterior bite for short duration if patient cooperation is a difficulty as seen in our case report.

## TECHNIQUES

1. Make impressions of upper arch and lower arches.
2. Make a posterior bite plate on upper arch with recommended extensions and sufficient enough for disocclusion.
3. Bond-brackets (0.18" or 0.22" slot, standard edgewise or preadjusted edgewise) on maxillary central incisors and insert round NiTi wire of smaller dimension (0.12" or 0.14" in 0.18" slot and 0.16" or 0.18" in 0.22" slot). Brackets are bonded more incisally than recommended.
4. Advise full time wear of posterior bite plate. Posterior bite plate should be cemented if patient cooperation is not anticipated.
5. Religate the NiTi wire and follow-up after each 3 weeks. Observe for patient discomfort if any and correction achieved.
6. Usually, one wire is sufficient and corrections are done in 6 to 9 weeks. Sever cases may require second wire. The second wire ligated is usually rectangular NiTi (0.16" × 0.22" in 0.18" slot and 0.18" × 0.25" in 0.22" slot) ligated in mesial tie wings only for better rotation correction.
7. After correction of rotation and leveling, remove posterior bite plane, bedond the twin brackets and cleanup the adhesive. Retainers are not usually recommended as crossbite correction is self retaining.

**Fig. 6:**
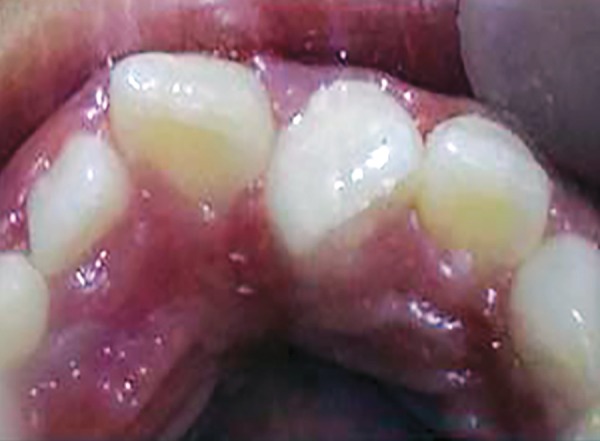
Intraoral maxillary occlusal view

**Fig. 7:**
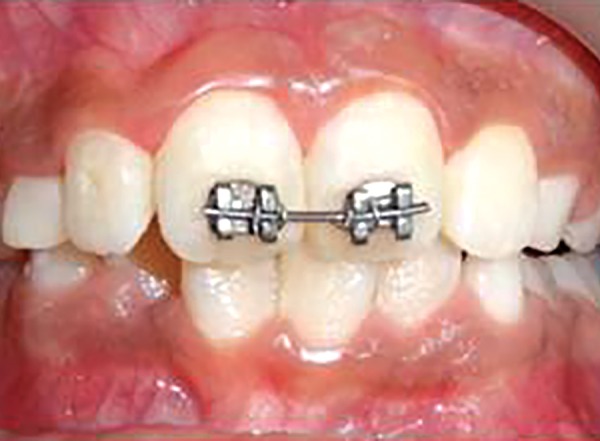
Second NiTi wire (0.16" × 0.22") ligated

**Fig. 8:**
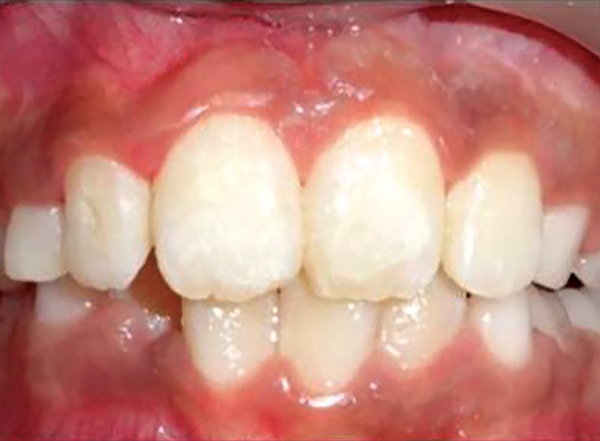
Intraoral frontal view after correction

**Fig. 9:**
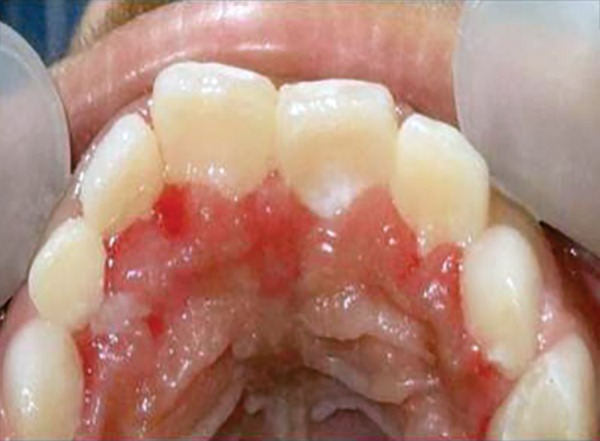
Intraoral maxillary occlusal view after correction

## CASE REPORT

**Fig. 10:**
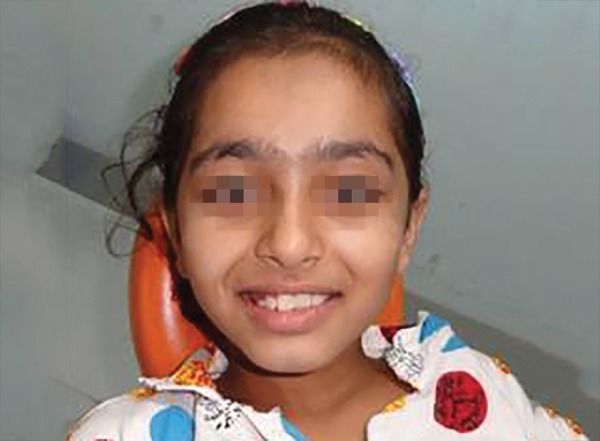
Smile of patient after crossbite correction

An eight and half years old Indian female patient born to nonconsanguineous parents in Meerut (Uttar Pradesh) reported to Department of Orthodontics and Dentofacial Orthopedics, Subharti Dental College, Meerut, with chief complaint of irregularly placed upper front teeth since 2 years. On examination, she was having straight profile, unesthetic smile. Intraoral examination revealed that she was in mixed dentition stage and maxillary left central incisor was found mesiolingually rotated and in cross-bite relation with mandibular incisors (Figs 5 and 6). Her orthopantomogram revealed that developing maxillary canines were close to the developing root apex of maxillary lateral incisors. Records were prepared. Based on her dental age, incisor correction was planned by anterior sectional twin bracket appliance. Posterior bite plane was constructed and cemented. Two preadjusted edgewise (MBT prescription) brackets were bonded on maxillary central incisors, and 0.14" NiTi wire was inserted (Fig. 4). Patient was given instruction regarding care of appliance and oral hygiene. Partial correction was seen at her first follow-up visit. On her second visit (sixth week), arch wire was changed to 0.16" × 0.22" NiTi (Fig. 7). Incisor came to its normal position on 3rd visit (9 weeks) as shown in Figures 8 and 9. Bite plane was removed, and brackets were debonded. Adhesive was removed, and teeth were polished. There was improvement in her smile (Fig. 10). She was followed up after 6 weeks as there was no evidence of relapse.

## CONCLUSION

Use of ASTBA for single tooth crossbite correction in selected cases is simple yet effective method. This method ensures excellent results in short duration even in uncooperative patients.
